# Chromosome Axis Defects Induce a Checkpoint-Mediated Delay and Interchromosomal Effect on Crossing Over during Drosophila Meiosis

**DOI:** 10.1371/journal.pgen.1001059

**Published:** 2010-08-12

**Authors:** Eric F. Joyce, Kim S. McKim

**Affiliations:** Waksman Institute and Department of Genetics, Rutgers, the State University of New Jersey, Piscataway, New Jersey, United States of America; The University of North Carolina at Chapel Hill, United States of America

## Abstract

Crossovers mediate the accurate segregation of homologous chromosomes during meiosis. The widely conserved *pch2* gene of *Drosophila melanogaster* is required for a pachytene checkpoint that delays prophase progression when genes necessary for DSB repair and crossover formation are defective. However, the underlying process that the pachytene checkpoint is monitoring remains unclear. Here we have investigated the relationship between chromosome structure and the pachytene checkpoint and show that disruptions in chromosome axis formation, caused by mutations in axis components or chromosome rearrangements, trigger a *pch2*-dependent delay. Accordingly, the global increase in crossovers caused by chromosome rearrangements, known as the “interchromosomal effect of crossing over,” is also dependent on *pch2*. Checkpoint-mediated effects require the histone deacetylase Sir2, revealing a conserved functional connection between PCH2 and Sir2 in monitoring meiotic events from *Saccharomyces cerevisiae* to a metazoan. These findings suggest a model in which the pachytene checkpoint monitors the structure of chromosome axes and may function to promote an optimal number of crossovers.

## Author Summary

Meiosis is a specialized cell division in which diploid organisms form haploid gametes for sexual reproduction. This is accomplished by a single round of replication followed by two consecutive divisions. At the first meiotic division, the segregation of homologous chromosomes in most organisms is dependent upon genetic recombination, or crossing over. Crossing over must therefore be regulated to ensure that every pair of homologous chromosomes receives at least one reciprocal exchange. Homologous chromosomes that do not receive a crossover frequently undergo missegregation, yielding gametes that do not contain the normal chromosome number, conditions frequently associated in humans with infertility and birth defects. The *pch2* gene is widely conserved and in *Drosophila melanogaster* is required for a meiosis-specific checkpoint that delays progression when crossover formation is defective. However, the underlying process that the checkpoint is monitoring remains unclear. Here we show that defects in axis components and homolog alignment are sufficient to induce checkpoint activity and increase crossing over across the genome. Based on these observations, we hypothesize that the checkpoint may monitor the integrity of chromosome axes and function to promote an optimal number of crossovers during meiosis.

## Introduction

Meiotic recombination occurs during prophase I when homologous chromosomes are synapsed along their entire length. Synapsis is defined as the close and stable association of homologous chromosomes through a proteinaceous structure called the synaptonemal complex (SC). In most organisms, this complex is composed of two main parts: lateral elements that attach along the axis of each homologous chromosome and transverse elements that span the central region of the SC and function to tether the homologs [1], [2]. At the leptotene/zygotene stages of meiotic prophase, these structural proteins begin to load onto the chromosome axes, and are completely assembled at pachytene, when homologous chromosomes are synapsed along their entire length.

Recombination between the homologous chromosomes initiates with DNA double-strand breaks (DSBs) that are repaired as either crossovers or noncrossovers [3]–[5]. Crossovers establish chromatin linkages called chiasmata, which, along with sister chromatid cohesion, hold homologs together after recombination has been completed and chromosomes have dissociated their SC proteins. Chiasmata help orient the homologous chromosomes on the metaphase I spindle and ensure their proper segregation at anaphase I. The failure to establish a crossover/chiasma can result in the nondisjunction of homologs and lead to aneuploid gametes.

Crossover formation is a tightly regulated process. Mutational analysis has revealed evidence for several mechanisms that control the frequency and position of crossovers along the chromosome arms [6]–[9]. For example, in *Drosophila melanogaster*, the precondition class of mutants exhibit reduced crossover levels with an altered distribution pattern, suggesting these genes have a role in establishing the number and distribution of crossover sites [10]. Changes in chromosome structure can also affect crossover distribution. Heterozygous inversions suppress crossing over near the breakpoints, yet enhance the frequency of exchange on the remaining chromosome pairs, a phenomenon referred to as the “interchromosomal effect” [11].

Crossing over may also be regulated by surveillance mechanisms that coordinate the sequence of critical events throughout prophase. In Drosophila, the process of repairing meiotic DSBs is monitored by at least two checkpoints: the canonical DSB repair checkpoint that responds to DNA damage [12], [13] and another that monitors DSB-independent events leading to crossover formation, hereafter referred to as the “pachytene checkpoint” [14]. The pachytene checkpoint induces a delay in response to defects in DSB repair genes required to repair all meiotic DSBs and genes encoding an endonuclease complex required for crossover formation (exchange class). Pachytene checkpoint activity requires a group of MCM-related genes that promote crossover formation (precondition class) and the Drosophila homolog of the widely conserved AAA+ ATPase PCH2.

In *Saccharomyces cerevisiae* and *Caenorhabditis elegans,* pachytene checkpoint activity has been detected in mutants with disrupted SC formation [15], [16]; however, it remains unclear what the underlying process is that the pachytene checkpoint is monitoring. For instance, yeast carrying a non-null *zip1* allele appear to form SC normally, yet still exhibit a Pch2-dependent delay [17]. Mutations that impair SC initiation in *C. elegans* triggers a Pch2-dependent response [16], although it is unclear whether the defect being monitored is synapsis *per se,* a prerequisite to synapsis such as homolog pairing and/or DSB repair. Some mutations that exhibit *pch2-*dependent delays in Drosophila have no obvious defects in SC formation and abolishing synapsis does not elicit any delay phenotypes [14]. Therefore, at least in Drosophila and possibly in these other organisms, it may not be the SC that is being monitored by the pachytene checkpoint. Instead, the pachytene checkpoint may be important to monitor synapsis-independent changes in chromosome structure required for crossover formation [14].

We have investigated the relationship between chromosome structure and the pachytene checkpoint and show that disruptions in chromosome axis components cause *pch2*-dependent delays. Unlike the delays observed in DSB repair mutants, these delays occur independently of MCM-related genes. Heterozygous chromosome aberrations also result in a MCM-independent pachytene delay and interchromosomal increase in crossovers that require *pch2*. These findings suggest a model in which the pachytene checkpoint monitors two genetically distinct events: an early function of DSB repair proteins and the structure of chromosome axes. A checkpoint response to both events requires the histone deacetlyase Sir2, showing that a functional connection between PCH2 and Sir2 in monitoring meiotic events is conserved in *Saccharomyces cerevisiae* and Drosophila. Checkpoint activity is also associated with prolonged PCH2 expression. We propose the pachytene checkpoint may function to promote an optimal number of crossovers by regulating the timing of a crossover determination phase defined by PCH2 expression.

## Results

### Defects in chromosome axis components cause a *pch2*-dependent pachytene delay

**Figure 1:**
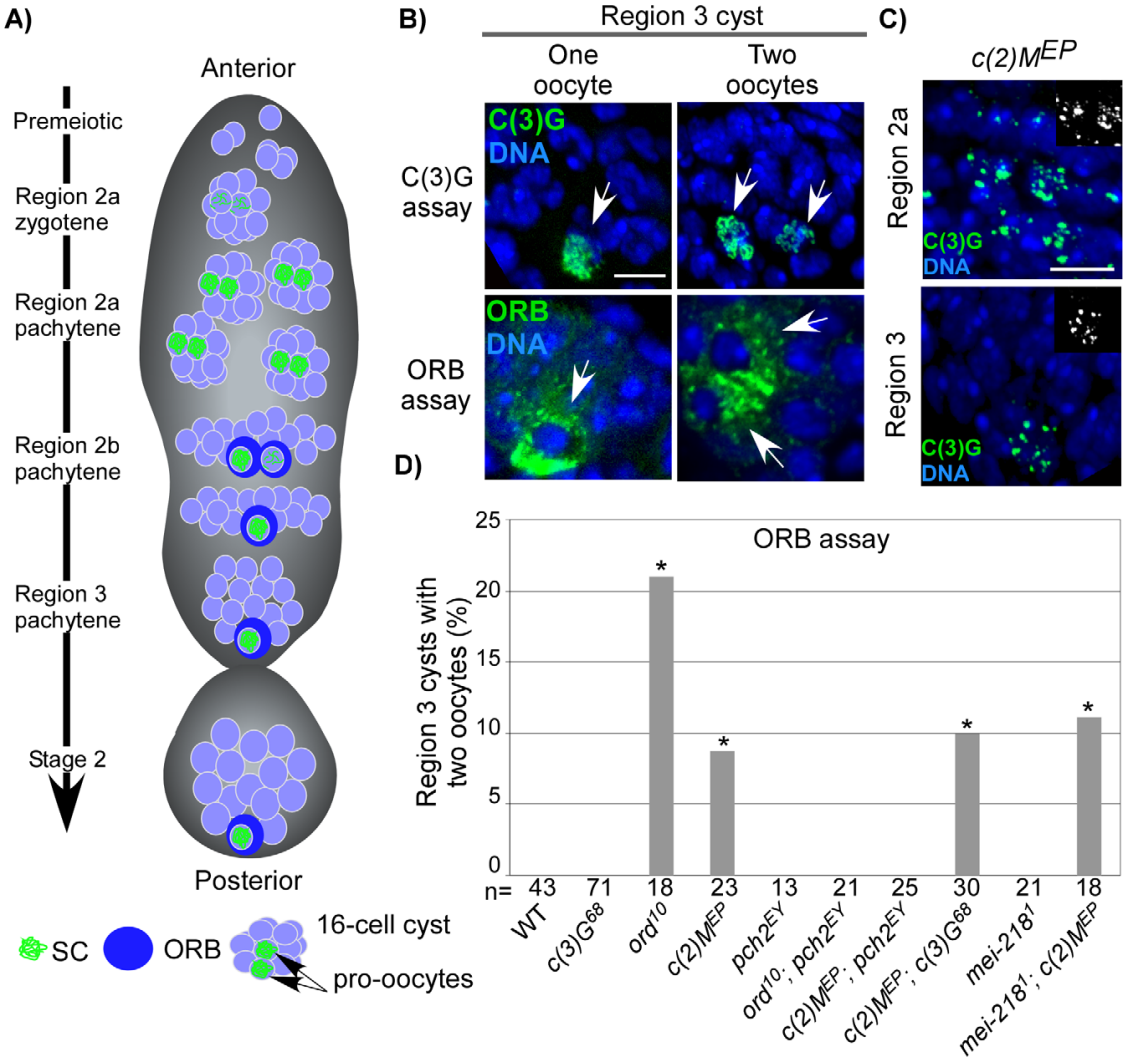
Pachytene delays in axis-defective mutants. (A) Schematic depiction of germline cysts and oocyte markers in a wild-type germarium. Changes in cyst morphology differentiate regions 2a (small and round cysts), 2b (flat oblong cysts), and 3 (large and round cysts). Cysts travel anterior to posterior and the age difference between each cyst is ∼12–24 hrs (our unpublished data and [56]). (B) Examples of region 3 cysts with one- or two-oocytes identified with C(3)G or the cytoplasmic ORB protein. Scale bar is 5 µM. (C) Threadlike C(3)G staining is never observed in the germaria of *c(2)M* mutants [52], which show fragments of C(3)G staining throughout pachytene, suggestive of an assembly defect. (D) ORB staining to detect oocytes in mutants with defective axis structure. A pachytene delay phenotype is defined as a significantly greater percentage of region 3 cysts with two-oocytes when compared to wild-type (asterisks are located above each bar when P-value was <0.05). Note that the pachytene delay observed in *ord* and *c(2)M* mutants was suppressed by mutation of *pch2* but not by mutation of *c(3)G* or *mei-218.* The number of cysts/germaria counted is at the bottom of each bar.

In the Drosophila germarium, oocytes are born within cysts composed of 16 cells that are connected by ring canals. Two out of the sixteen cells, each with four ring canals, initially contain equivalent levels of SC proteins and are termed the pro-oocytes (Figure 1A). As the developing cysts travel from the anterior (region 2) toward the posterior (region 3) of the germarium, the pro-oocytes proceed through the pachytene stage of meiosis where synapsis is completed and DSB formation and recombination occurs. By region 3 of the wild-type germarium, DSB repair is completed and one of the two pro-oocytes will exit meiosis, lose its SC and become a nurse cell while the other will continue through development and form the oocyte (Figure 1A) [18].

In DSB repair and exchange-defective mutants, the transition through pachytene is delayed by pachytene checkpoint activity [14]. This results in both pro-oocytes persisting into region 3 cysts, referred to as the “two-oocyte phenotype.” Delays can be identified either by the persistence of the SC transverse filament C(3)G in both pro-oocytes [14] or by the concentration of ORB protein in the cytoplasm of two cells rather than one in region 3 cysts (Figure 1B) [19]. ORB staining, however, is less sensitive than C(3)G at detecting pachytene delays, resulting in a different frequency of the two-oocyte phenotype between the two assays [14].

Abolishing synapsis by mutation of *c(3)G* does not elicit the two-oocyte phenotype (Figure 1D), suggesting the pachytene checkpoint is not monitoring SC formation [14]. We further investigated the relationship between chromosome structure components and the pachytene checkpoint by determining the effects of mutations in two other genes, *ord* and *c(2)M,* which encode structural proteins.

ORD localizes to chromosome axes in oocytes independent of synapsis (i.e. in *c(3)G* mutants) and has roles in meiotic recombination and sister chromatid cohesion [20], [21]. Although *ord* mutants initially display normal C(3)G and C(2)M localization, only rare structures resembling SC were observed by electron microscopy (EM), suggesting that the ultrastructure of chromosome axes was disorganized [21]. Consistent with this interpretation, C(3)G and C(2)M staining precociously deteriorates in *ord* mutants as the oocytes progress through pachytene [21]. We found that *ord* mutants displayed a high frequency of the two-oocyte phenotype (Figure 1D), indicative of a delay in meiotic progression. The two-oocyte phenotype was suppressed in *ord; pch2* double mutants, indicating the delay was dependent on the pachytene checkpoint (Figure 1D) and supporting the hypothesis that the pachytene checkpoint is sensitive to defects in axis components.

C(2)M is a component of the SC lateral element and localizes adjacent to the chromosome axes even in the absence of synapsis (in *c(3)G* mutants), suggesting it may interact with axis components [22], [23]. In *c(2)M* mutant oocytes, C(3)G protein fails to develop into complete strands along the lengths of each chromosome, but instead appears as small patches (Figure 1C). The most likely explanation is that SC initiates in *c(2)M* mutants but polymerization is defective. Similar to *ord* mutants, *c(2)M* mutants exhibited a high frequency of the two-oocyte phenotype, which was suppressed in *c(2)M; pch2* double mutants (Figure 1D). The high frequency of the two-oocyte phenotype observed in *c(2)M* mutants was not suppressed by mutation of *c(3)G* (Figure 1D), demonstrating the pachytene checkpoint can signal independently of SC initiation. Together, these results suggest the pachytene checkpoint may monitor a synapsis-independent function of ORD and C(2)M, such as the formation of chromosome axes.

### Chromosomal rearrangements disrupt axis integrity and cause a *pch2*-dependent pachytene delay

**Figure 2:**
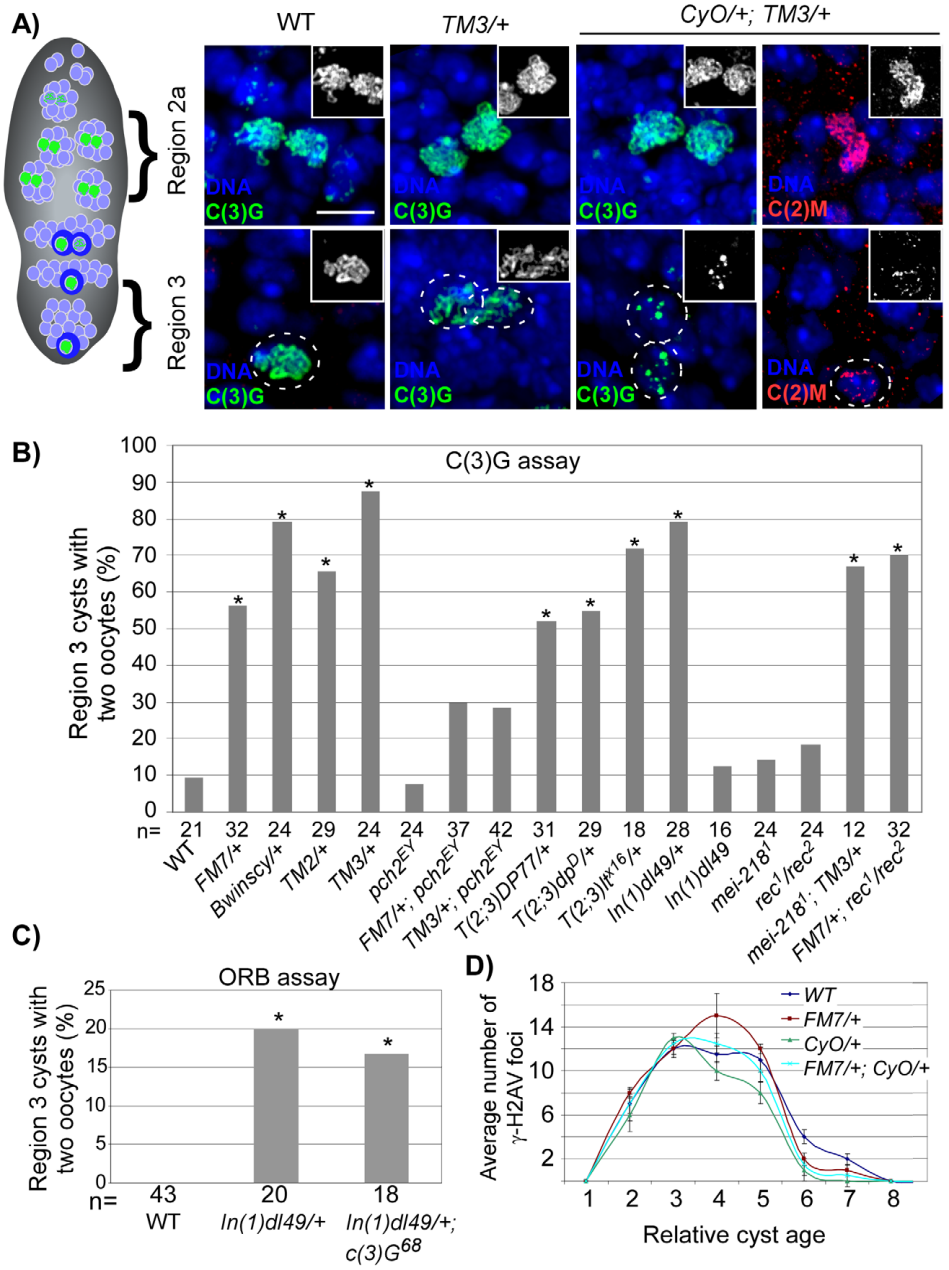
SC deterioration and pachytene delays in balancer heterozygotes. (A) SC localization in region 2 and region 3 cysts from wild-type, *TM3/+,* and *CyO/+; TM3/+* females. Oocytes in region 3 are outlined. In wild-type and *TM3/+* germaria, extensive threadlike C(3)G typical of pachytene is visible in oocytes throughout the germarium. In *CyO/+; TM3/+* germaria, threadlike C(3)G and C(2)M staining is detected in region 2a, but deteriorates in the majority of region 3 oocytes. Scale bar is 5 µM. (B) Based on C(3)G staining, a high frequency of the two-oocyte phenotype was found in heterozygotes for single translocations [*T(2;3)*], a single inversion [*In(1)d149*] and the balancer chromosomes *FM7, Bwinscy, TM2,* and *TM3.* This was suppressed by mutation of *pch2,* but not by *mei-218.* Asterisks are located above each bar when P-value was <0.05 compared to wild-type. The number of cysts counted is at the bottom of each bar. (C) Based on ORB staining, the high frequency of the two-oocyte phenotype in *In(1)dl49/+* heterozygotes was not suppressed by mutation of *c(3)G*. (D) The average number of γ-H2AV foci as a function of relative cyst age in wild-type and balancer heterozygotes. Cyst 1 is the first to have complete SC, cyst 8 is in region 3 and cysts 9–11 are in later-stage cysts (stages 2–4), which have left the germarium. Neither single nor double balancer heterozygotes significantly altered the appearance or disappearance of γ-H2AV foci throughout the germarium. Error bars denote the standard error of the mean.

If the pachytene checkpoint monitors the integrity of chromosome axes we reasoned that structural rearrangements would also exhibit pachytene delays. Balancers are multiply-inverted chromosomes that fail to cross over with a normal homolog, presumably due to a disruption in the continuity of pairing and/or synapsis [24]–[26]. We characterized the integrity of SC-associated proteins in balancer heterozygotes with antibodies recognizing the SC components C(3)G and C(2)M. Single balancer heterozygotes (*TM3/+*) had thread-like C(3)G and C(2)M staining that was indistinguishable from wild-type (Figure 2A) [26]. Double balancer heterozygotes (*CyO/+; TM3/+*)also initially displayed normal C(3)G and C(2)M localization, but the staining became fragmented and sometimes undetectable in region 3 oocytes (Figure 2A). This precocious deterioration of SC proteins during pachytene is similar to what is observed in *ord* mutant oocytes [21], suggesting that rearrangement breakpoints might disrupt axis stability.

Using C(3)G staining to detect oocytes, we found that *FM7, Bwinscy, TM2* and *TM3* balancer heterozygotes each exhibited a significantly higher frequency of the two-oocyte phenotype compared to wild-type (Figure 2B), suggestive of a pachytene delay. The high frequency of the two-oocyte phenotype was suppressed to wild-type levels in *FM7/+; pch2* and *TM3/+; pch2* females, confirming the delays were dependent on the pachytene checkpoint (Figure 2B; P<0.05 compared to either balancer heterozygote alone). *pch2* had no effect on the SC morphology in single balancer heterozygotes (data not shown).

Each balancer chromosome contains several inversions. For example, the *TM3* chromosome includes 10 breakpoints [27]. To investigate the effects of a more subtle disruption in chromosome organization on the pachytene checkpoint, we tested whether a single aberration, or two breakpoints, would be enough to induce pachytene delays. Remarkably, heterozygotes of single translocations between the 2^nd^ and 3^rd^ chromosomes (*T(2;3)DP77/+, T(2;3)dp^D^/+,* and *T(2;3)lt^X16^/+*) and a single inversion on the X chromosome (*In(1)dl49/+*) each exhibited a high frequency of the two-oocyte phenotype, suggesting the threshold to trigger the pachytene checkpoint is low and requires as few as two breaks in axis continuity (Figure 2B). Importantly, the delays were not dependent on C(3)G and were not significantly increased in *In(1)dl49* homozygotes compared to wild-type (Figure 2B and 2C), indicating the pachytene checkpoint responds to a break in alignment between homologs in a way that is independent of SC initiation.

### Chromosome axis defects cause pachytene checkpoint delays independent of the MCM–related precondition genes

In addition to the delay in oocyte selection, DSB repair and exchange-defective mutants also exhibit a *pch2*-dependent delay in the response to DSBs [14]. To monitor DSB formation and repair in balancer heterozygotes, we stained ovaries with an antibody to γ-H2AV [28], [29]. In wild-type oocytes, γ-H2AV foci are most abundant in region 2a nuclei (cyst 3 in Figure 2D) and absent by region 3 (cyst 8 in Figure 2D), indicating DSBs have been repaired. Likewise, both *FM7/+* and *CyO/+* heterozygotes exhibited maximum γ-H2AV foci in region 2a oocytes at a similar cyst age to wild-type (Figure 2D). The same result was also observed in the double heterozygote *FM7/+; CyO/+*. Therefore, balancer heterozygotes do not cause a delay in the γ-H2AV response to DSBs, revealing a distinction between the effect of DSB repair mutants and chromosomal rearrangements on the pachytene checkpoint. While mutations in DSB repair genes induce two *pch2*-dependent delays in pachytene, delayed response to DSBs and delayed oocyte selection, chromosomal rearrangements only delay the latter.

If the pachytene checkpoint can cause delays through two distinct pathways, it should be possible to define them genetically. This was tested with mutations in the MCM-related precondition genes *mei-218* and *rec,* which are required for 90% of all crossovers and the pachytene delays caused by mutations in DSB repair and exchange genes [14]. Unexpectedly, the high frequency of the two-oocyte phenotype was still observed in *mei-218; TM3/+* and *FM7/+; rec* (Figure 2B, P<0.05 compared to *mei-218* and *rec* single mutants). Consistent with this finding, the pachytene delay in *c(2)M* mutants was not suppressed in *mei-218; c(2)M* double mutants (Figure 1D). These results show that, unlike the DSB repair and exchange-defective mutants, defective and/or misaligned chromosome axes interact with the pachytene checkpoint independent of precondition genes and possibly at a later step (*i.e.* after the DSB response).

### PCH2 can induce interchromosomal effects on crossing over

**Figure 3:**
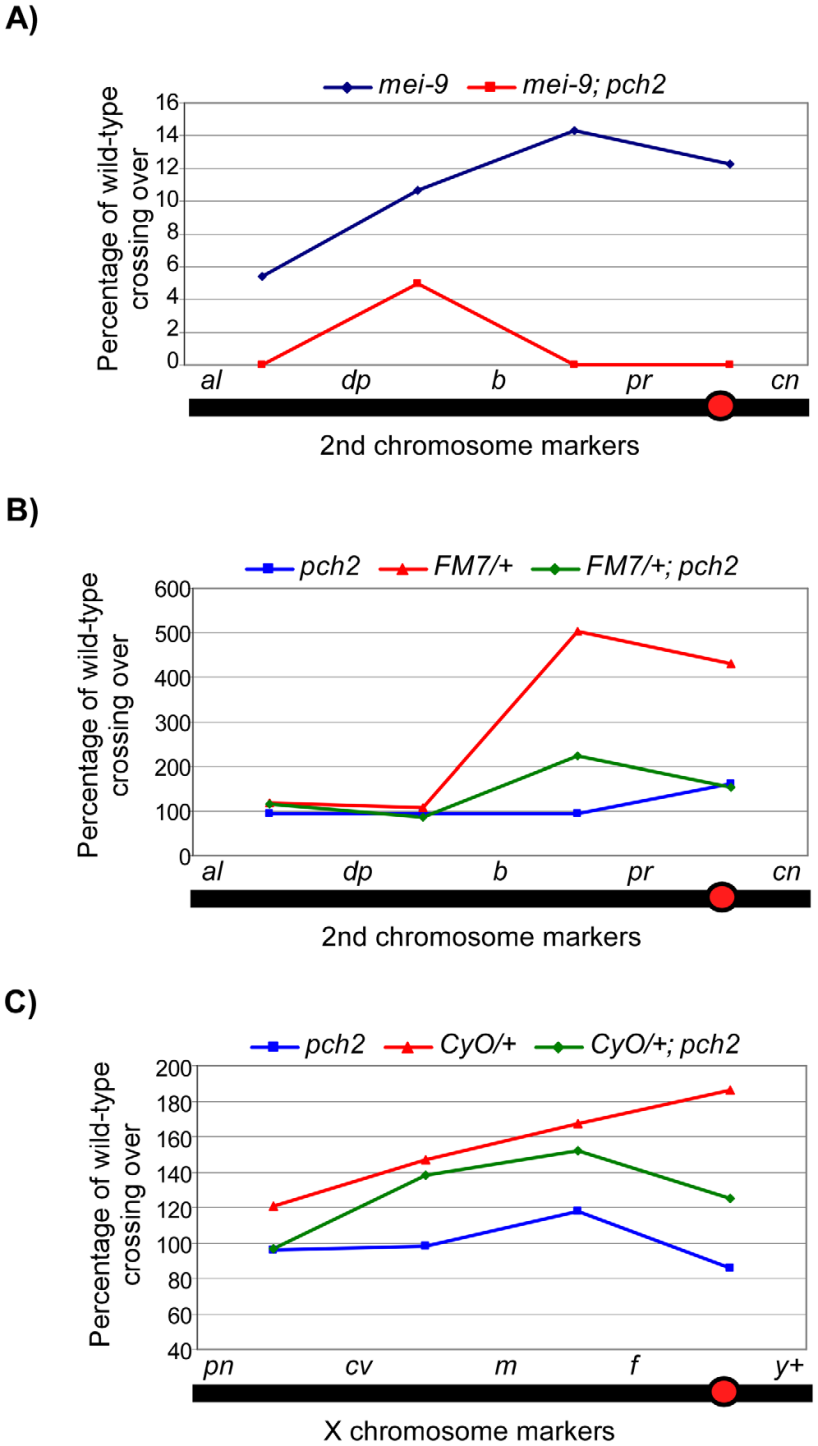
Percentage of wild-type crossing over in balancer heterozygotes, *mei-9*, and *pch2* mutant females. (A) Percentage of wild-type crossing over in *mei-9* and *mei-9; pch2* mutant females along the 2^nd^ chromosome. Most crossovers in a *mei-9* mutant are dependent on *pch2* (also see Table 1). (B) Percentage of wild-type crossing over along the 2^nd^ chromosome in *pch2*, *FM7/+*, and *FM7/+; pch2* mutant females. *pch2* mutants have wild-type levels and distribution of exchange. The interchromosomal effect of the *FM7* balancer was mostly suppressed in *pch2* mutants (also see Table 1). (C) Percentage of wild-type crossing over along the X-chromosome in *pch2*, *CyO/+*, and *CyO/+; pch2* mutant females (note the scale is reduced). The interchromosomal effect elicited by the *CyO* balancer was reduced in *pch2* mutants, especially in the most proximal and distal intervals (also see Table 2).

PCH2 is required for some of the crossovers that occur in the exchange-defective mutant, *hdm* [14]. Consequently, *hdm; pch2* double mutants exhibit an elevated frequency of nondisjunction compared to *hdm* single mutants. These results suggest a functional link between the pachytene checkpoint and the production of crossovers. To determine if this is a general property of mutants that exhibit pachytene delays, we tested additional double mutants with *pch2.* Exchange class genes *Ercc1* and *mei-9* encode components of an endonuclease complex of proteins that includes HDM and is required for normal levels of meiotic crossing over [30], [31]. Loss of ERCC1 function results in 14% X-chromosome nondisjunction, which is elevated to 30% in a *pch2* mutant background, suggesting crossovers are further reduced (Table S1). In addition, the low level of crossovers that are generated along the 2^nd^ chromosome in *mei-9* mutants are mostly suppressed in *mei-9*; *pch2* double mutants (Figure 3A). These results suggest the residual crossovers in recombination-defective mutants depend on a mechanism facilitated by *pch2*.

**Table 1:** Effect of *pch2* on crossing over on the 2^nd^ chromosome.

	Crossing over on the Second Chromosome (cM)^a^					
Genotype^b^	*al-dp*	*dp-b*	*b-pr*	*pr-cn*	Total *al-cn*	N^c^
Wild-type	12.5	28.0	3.5	1.3	45.3	1008
*pch2^EY^*	11.7 (94)	26.7 (95)	3.3 (94)	2.1 (162)	43.8 (97)	562
*mei-9* a	0.67 (5.4)	3.0 (10.7)	0.5 (14.3)	0.2 (12.3)	4.3 (9.5)	993
*mei-9* a *; pch2^EY^*	0.0 (0.0)	1.4 (5.0)	0.0 (0.0)	0.0 (0.0)	1.4 (3.1)	360
*FM7/+*	14.8 (118)	30.3 (108)	17.6 (503)	5.6 (431)	68.3 (151)	568
*FM7/+; pch2^EY^*	14.6 (117)	23.8 (85)	7.8 (223)	2.0 (154)	48.2 (106)	1176
*MVD1 P(HA-pch2)27/+*	12.0 (96)	36.0 (129)	5.5 (157)	0.9 (69)	54.4 (120)	1300
*MVD1 P(HA-pch2)71/+*	11.5 (92)	25.0 (89)	7.5 (214)	1.3 (100)	45.3 (100)	1404
*MVD1 P(HA-pch2)81/+*	9.3 (74)	31.5 (113)	6.5 (186)	1.6 (123)	48.9 (108)	992
*MVD1 P(HA-pch2)71*	9.8 (78)	41.7 (149)	10.3 (293)	0.8 (60)	62.6 (138)	1026

a Second chromosome crossing over was assayed by crossing *al dp b pr cn/+* females to *al dp b pr cn/CyO* males in the indicated backgrounds. The *Cy^+^* progeny were scored for recombinants. Crossing over is expressed as cM across the intervals shown. Numbers in parentheses denote the percentage of wild-type recombination frequency.

b *MVD1* =  *P(Gal4::VP16-nos.UTR)MVD1*.

c N =  total flies counted.

**Table 2:** Effect of *pch2* on crossing over on the X-chromosome.

	Crossing over on the X Chromosome (cM)^a^					
Genotype	*pn-cv*	*cv-m*	*m-f*	*f-y^+^*	Total *pn-y^+^*	N^b^
Wild-type	15.4	18.9	11.9	7.3	53.5	657
*pch2^EY^*	14.8 (96)	18.5 (98)	14.1 (118)	6.3 (86)	53.7 (100)	569
*CyO/+*	18.6 (121)	27.7 (147)	19.9 (167)	13.6 (186)	79.8 (149)	1319
*CyO/+; pch2^EY^*	15.0 (97)	26.1 (138)	18.1 (152)	9.1 (125)	68.3 (128)	1148

a X chromosome crossing over was assayed by crossing *y pn cv m f • y^+^/y* females to wild-type males in the indicated backgrounds. The male progeny were scored for recombinants. Crossing over is expressed as cM across the intervals shown. Numbers in parentheses denote the percentage of wild-type recombination frequency.

b N =  total flies counted.

When crossing over is suppressed along a normal chromosome heterozygous to a balancer, there is an interchromosomal effect that increases crossing over on the remaining chromosome pairs [11]. Since PCH2 is responsible for the residual level of crossovers in recombination-defective mutants, we asked if it was also responsible for the increase in crossovers observed in balancer heterozygotes. Consistent with previous work on interchromosomal effects [32], [33], we found that *FM7/+* heterozygotes exhibit 151% of wild-type crossing over along the 2^nd^ chromosome with an altered distribution (Table 1). Although there was little deviation from wild-type controls in the distal regions of the chromosome (*al-b*), the genetic map distance was increased ∼4–5 times that observed in wild-type centromere-proximal intervals (Table 1; Figure 3B). Remarkably, 2^nd^ chromosome crossing over in *FM7/+* heterozygotes was reduced to 106% of wild-type in a *pch2* mutant background (p<0.00005; Table 1; Figure 3B). Similarly, introduction of the *CyO* chromosome increased crossing over along the X chromosome to 149% of wild-type, which was reduced to 128% in *pch2* mutants (p<0.05; Table 2; Figure 3C). Interestingly, the closer the interval being tested was to the centromere, the greater the interchromosomal effect and *pch2* dependence (Table 2; Figure 3C). Since *pch2* single mutants exhibited normal levels of crossing over on the X and 2^nd^ chromosome (Table 1; Table 2; Figure 3), these data show that *pch2* is required for most of the interchromosomal increase in crossover levels in balancer heterozygotes.

### Pachytene checkpoint activity does not lead to an increase in DSB levels

The increased crossing over observed in balancer heterozygotes could be explained by pachytene checkpoint activity increasing DSB levels. However, we failed to observe any significant change in the number of γ-H2AV foci in oocytes single or doubly heterozygous for *FM7* and *CyO* compared to wild-type (Figure 2D). Since asynchrony of DSB formation can complicate measuring the total number of γ-H2AV foci, we repeated the above experiment in a *spn-A* (Drosophila Rad51) mutant background, in which repair of DSBs is blocked [12]. The number of γ-H2AV foci in region 3 oocytes of these mutants is expected to be close to the total number of DSBs that occurred [28], [34]. *spn-A* mutant region 3 oocytes displayed an average of 21.0 (+/−1.5) γ-H2AV foci. Similarly, *FM7/+; CyO/+; spn-A* region 3 oocytes had an average of 24.0 (+/−1.4) γ-H2AV foci.

These results indicate that the ability of the pachytene checkpoint to increase crossing over in the genome is not mediated by a substantial increase in the total number of DSBs. Instead, pachytene checkpoint activity most likely increases the chance of DSBs becoming crossovers, particularly those that occur near centromeres.

### PCH2 localizes to the nuclear periphery and persists when pachytene is delayed

To investigate how PCH2 affects crossover frequency, we monitored the protein localization pattern during meiosis. A transgene was constructed containing a hemagluttin (HA) epitope at the N-terminus of the *pch2* transcript under the control of an inducible UASP promoter. We expressed PCH2 using the germline specific driver *P(Gal4-nos.NGT)40* [35], abbreviated as *NGT,* known to express in pachytene at moderate levels [36]. The *NGT*-driven *P(HA-pch2)71* transgenic line restored the two-oocyte phenotype in *FM7/+; pch2* females to similar levels found in *FM7/+* heterozygotes (Figure S1), demonstrating that the *NGT*-driven *pch2* transgene was functional.

**Figure 4:**
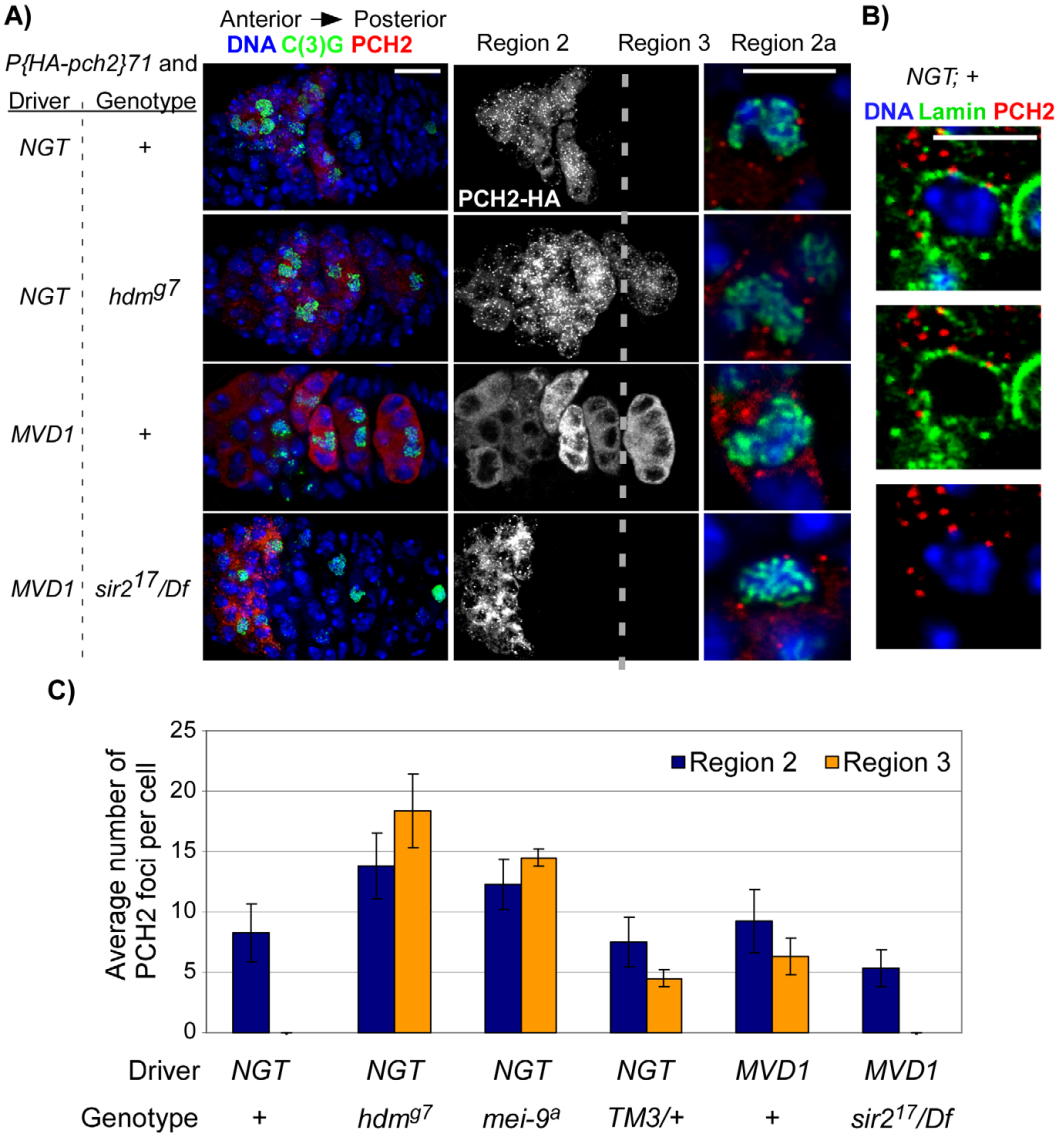
PCH2 localization during pachytene. (A) PCH2 localization in germaria when the *UASP: pch2* transgene was driven by *P(Gal4-nos.NGT)40* (* = NGT*) or by *P(Gal4::VP16-nos.UTR)MVD1* (* = MVD1*). Single sections of region 2a oocytes are shown to the right of their corresponding germarium. Scale bars for germaria and single cells are 10 µM and 5 µM, respectively. In controls, PCH2 expression is restricted to zygotene and early pachytene cells (region 2). PCH2 expression persists into region 3 cells of *hdm* mutants and when PCH2 is driven by *MVD1.* In *MVD1*-driven PCH2, two populations of PCH2 become present: unlocalized protein distributed evenly throughout the cytoplasm and distinct bright spots classified as foci. Due to the projection of multiple images, it is difficult to see PCH2 foci among the unlocalized staining in the *MVD1; +* germarium. They are visible in single sections. PCH2 expression in region 3 cells is eliminated in *sir2* mutants. PCH2 foci localize adjacent to the DNA stain in all genotypes. (B) When the *UASP: pch2* transgene was driven by *P(Gal4-nos.NGT)40,* PCH2 foci in region 2a oocytes localize to the cytoplasmic side of the nuclear envelope protein, Lamin. (C) Quantification of PCH2 foci. The average number of PCH2 foci per cell was increased in both region 2 and region 3 cells of *hdm* and *mei-9* mutants. PCH2 foci levels did not increase in *TM3* heterozygotes or when PCH2 was overexpressed by the *MVD1* driver, but did persist into region 3 cells.

PCH2 staining formed foci that localized around the nucleus in zygotene and early pachytene (region 2) cells (Figure 4A). No PCH2 foci were detected in region 3 cells, suggesting the protein is rapidly degraded or no longer produced after early pachytene. Surprisingly, PCH2 foci did not overlap with the DNA stain. To determine if PCH2 foci localized within the nucleus, we counterstained with the nuclear envelope component, Lamin. We found that 73% of PCH2 foci showed a close association (i.e. touching) with the cytoplasmic side of the Lamin staining (n = 368; Figure 4B), indicating they localized adjacent to the nuclear envelope and outside the nucleus. The remaining 27% of PCH2 foci not closely associated with Lamin were found dispersed within the cytoplasm.

To determine if PCH2 localization pattern changes in mutant backgrounds that exhibit pachytene delays, we examined PCH2 expression in mutants that cause checkpoint delays: *hdm, mei-9* and in *TM3/+* heterozygotes. In *hdm* and *mei-9* mutants, the number of PCH2 foci per oocyte was increased ∼2-fold compared to controls (Figure 4C). In addition, the foci persisted into region 3 oocytes, which was never observed in control germaria (Figure 4A and 4C). However, PCH2 localization was not detected past stage 2 of oogenesis (data not shown), indicating the loss of PCH2 is only delayed in exchange-defective mutants. In *TM3/+* heterozygotes, the levels of PCH2 foci in region 2 cells was unchanged compared to controls, but were present in region 3 (Figure 4C), revealing a correlation between the prolonged expression of PCH2 and a delay in pachytene.

The intracellular localization pattern of PCH2 did not change when pachytene was delayed since the foci remained juxtaposed to the nuclear envelope in *hdm* and *mei-9* mutants and in *TM3/+* heterozygotes at all stages (Figure 4A and data not shown). Furthermore, mutation of *mei-W68,* which eliminates DSB formation, showed a normal staining pattern of PCH2, and *hdm; mei-W68* double mutants showed the same PCH2 staining pattern as *hdm* single mutants (Figure 4A and data not shown), consistent with our previous conclusion that the pachytene checkpoint functions independently of DSB formation [14], [16]. These observations provide a connection between the nuclear envelope and pachytene checkpoint activity and suggest that PCH2′s role in nuclear events like crossing over is indirect and at a distance from the chromosomes.

### Prolonged PCH2 activity leads to a pachytene delay and altered crossover distribution

To test the significance of the correlation between pachytene delays and prolonged PCH2 expression, we manipulated the timing and expression levels of PCH2 in the germline. PCH2 levels were increased by driving the *UASP:pch2* transgene with *P(Gal4::VP16-nos.UTR)MVD1* [37], abbreviated as *MVD1,* known to drive high levels of expression in the germarium. *MVD1*-driven *pch2* caused the protein to persist into region 3 oocytes, which was never observed with the *NGT* driver in a wild-type background (Figure 4A and 4C). In addition to distinct foci, PCH2 was also distributed more evenly throughout the cytoplasm (Figure 4A). Thus, *MVD1*-driven *pch2* resulted in overproduction and prolonged expression of the protein throughout pachytene.

**Figure 5:**
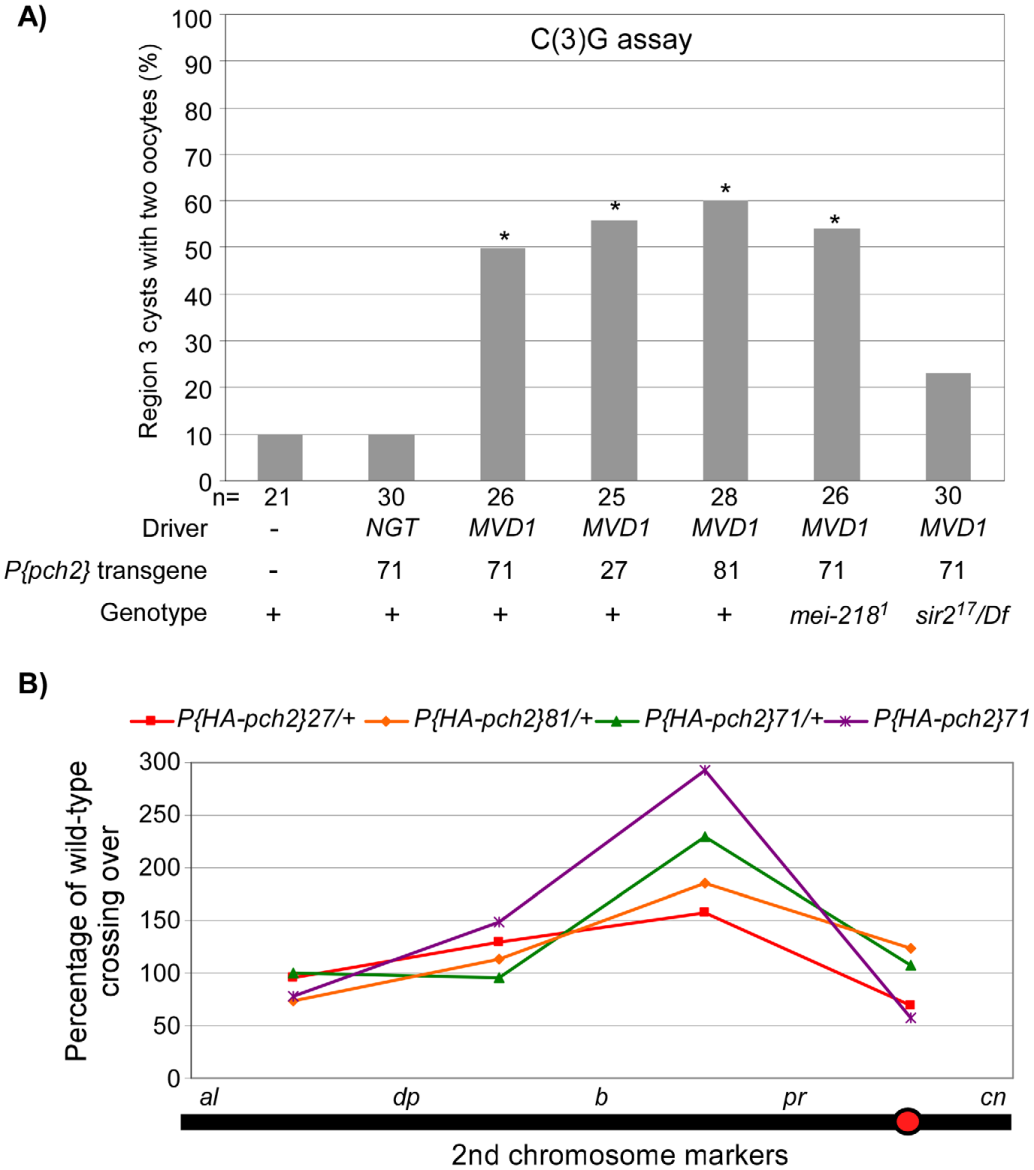
PCH2 overexpression leads to pachytene delays and altered crossover distribution. (A) Three different PCH2 transgenic lines driven by *MVD1* exhibit a high frequency of the two-oocyte phenotype whereas PCH2 driven by *NGT* does not. The pachytene delay in *MVD1*-driven PCH2 was suppressed by mutation of *sir2*, but not by *mei-218* (also see Figure S1). Asterisks are located above each bar when P-value was <0.05 compared to wild-type. The number of cysts counted is at the bottom of each bar. (B) Percentage of wild-type crossing over along the 2^nd^ chromosome in three different transgenic lines where PCH2 is overexpressed by the *MVD1* driver. All lines display a similar altered distribution pattern with elevated exchange in the *b-pr* interval, yet each exhibits a different level of effect on total crossover levels. Two copies of the transgene driven by *MVD1* have the greatest effect on both crossover distribution and levels.

Pachytene delays were not observed when the *pch2* transgenes were expressed using the *NGT* driver (Figure 5A). In contrast, *MVD1*-driven *pch2* induced a pachytene delay that resulted in a high frequency of the two-oocyte phenotype (Figure 5A). In fact, 100% (n = 10) of the germaria with PCH2 expression in region 3 cysts also contained two oocytes, as viewed by C(3)G staining, suggesting prolonged PCH2 expression is sufficient to induce a delay in pachytene progression.

Since overproducing PCH2 caused a pachytene delay, we determined if crossover frequency or distribution was also affected. We found that PCH2 expression driven by *MVD1* altered the distribution of exchange in all 3 transgenic lines tested (Table 1; Figure 5B). The most dramatic increase in crossover frequencies was observed in the centromere proximal interval of chromosome 2, *b-pr.* Although all the transgenic lines that were tested showed the same change in crossover distribution, the magnitude was different, which probably reflects different transgenic protein levels. In support of this conclusion, the presence of two transgenic copies of *P(HA-pch2)71* driven by *MVD1* exacerbated the effect on both crossover levels and distribution (Table 1; Figure 5B). These data show that the frequency and distribution of crossing over is sensitive to the timing and level of PCH2 expression during pachytene.

### *sir2* is required for prolonged PCH2 expression and the pachytene checkpoint

We sought to identify factors that facilitate prolonged PCH2 expression and cause pachytene delays. The first candidate we tested was *mei-218* since it is required for the *pch2*-dependent pachytene delays observed in DSB repair and exchange-defective mutants. *mei-218* mutants, however, did not show an effect on the levels or distribution of *MVD1*-driven PCH2 (Figure S2). Also, the two-oocyte phenotype caused by PCH2 overexpression was not suppressed in *mei-218* mutants (Figure 5A), suggesting MEI-218 is not required for PCH2 localization.

The second candidate tested was Sir2, which encodes a histone deacetylase that is required for the nucleolus localization of Pch2 and the pachytene checkpoint during *S. cerevisiae* meiosis [15]. Five Drosophila genes belong to the Sir2 family. Of these, Sir2 is the closest homolog of the *S. cerevisiae* Sir2 [38]. Drosophila *sir2* null alleles have no obvious effects on viability, but affect position effect variegation, heterochromatic silencing and fly life span [38]–[40]. *sir2* mutants were fully fertile with wild-type levels of X-chromosome nondisjunction (Table S1), indicating Sir2 is dispensable for meiotic recombination.

**Figure 6:**
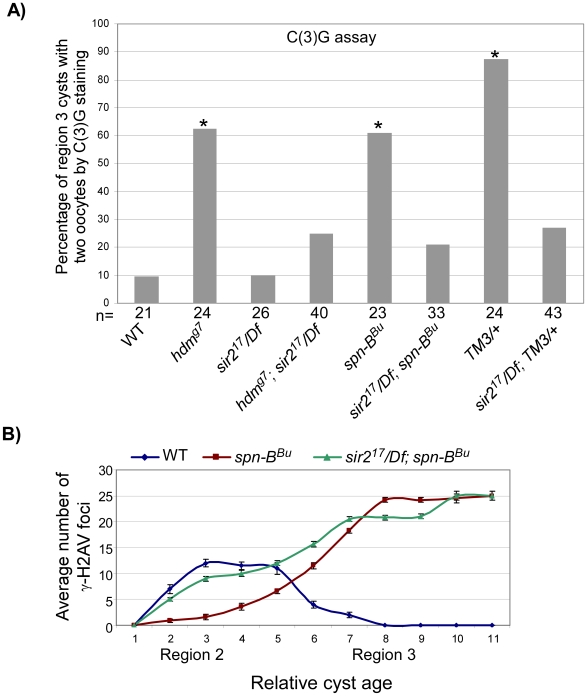
*sir2* is required for the pachytene delays. (A) Mutation of *sir2* suppressed the high frequency of the two-oocyte phenotype observed in the exchange-defective mutant *hdm,* DSB repair mutant *spn-B* and in *TM3* heterozygotes. Asterisks are located above each bar when P-value was <0.05 compared to wild-type. The number of cysts counted is at the bottom of each bar. (B) The average number of γ-H2AV foci is plotted as a function of relative cyst age in wild-type, *spn-B* and *sir2; spn-B* mutants. Mutation of *sir2* suppressed the delayed onset of γ-H2AV (see cyst 2–5) in *spn-B* mutants. *spn-B* mutants also have a block in DSB repair that cause γ-H2AV to accumulate into late stages of oogenesis, which is not suppressed by *sir2*. Error bars denote the standard error of the mean.

We investigated whether Sir2 is involved in the pachytene checkpoint and found that mutation of *sir2* suppressed the high frequency of the two-oocyte phenotype observed when PCH2 is overexpressed with the *MVD1* driver (Figure 5A). The high frequency of the two-oocyte phenotypes observed in the exchange-defective mutant *hdm* and DSB repair mutant *spn-B* were also suppressed by *sir2* (Figure 6A). Likewise, Sir2 was required for the pachytene delay observed in *TM3/+* heterozygotes (Figure 6A) and the delayed onset of γ-H2AV staining in *spn-B* mutants (cyst 2–5 in Figure 6B). Thus, like *pch2*, *sir2* is required for the pachytene checkpoint.

Strikingly, the region 3 localization of *MVD1*-driven PCH2 was eliminated in a *sir2* mutant (Figure 4A and 4C). In contrast, loss of *sir2* only slightly reduced the level of PCH2 in region 2 cells and had no effect on the peri-nuclear localization of PCH2 driven by *NGT* (Figure 4C and data not shown). In addition, expression of a *c(2)M* transgene driven by *MVD1* was not affected, indicating the effect of *sir2* on PCH2 was not due to a reduction in the transcription of UAS-driven genes (Figure S3). These results support the hypothesis that high levels of PCH2 are dependent on Sir2 and essential for the pachytene delays.

## Discussion

### The pachytene checkpoint is sensitive to defects in chromosome axes

We have previously shown that removing the SC central element component C(3)G does not cause *pch2*-dependent delays in Drosophila meiotic prophase [14]. Therefore, the pachytene checkpoint is not monitoring the process of synapsis *per se*. Instead, two lines of evidence suggest the pachytene checkpoint is sensitive to defects in chromosome axes. First, mutations in genes that encode structural axis components, C(2)M and ORD, cause *pch2*-dependent pachytene delays. Second, heterozygous chromosomal rearrangements also cause a *pch2*-dependent delay. Homozygous rearrangements do not cause delays; therefore, the pachytene checkpoint is sensitive to any discontinuity in the alignment between homologous chromosomes. Since the delays do not depend on C(3)G, the defect must be prior to or independent of synapsis initiation. The misalignment of homologous sequences could destabilize the integrity of chromosome axes, such as the assembly of ORD or C(2)M, and expose substrates that trigger the pachytene checkpoint. Indeed, females doubly heterozygous for balancer chromosomes show deterioration of C(2)M staining in pachytene oocytes similar to *ord* mutants [21], suggesting the axial elements are compromised by the heterozygous inversion breakpoints.

### Two genetically distinct pathways can trigger the pachytene checkpoint

The delays observed in *c(2)M* mutants and balancer heterozygotes do not depend on the MCM-related precondition genes such as *mei-218,* which are required for the pachytene delays in DSB repair and exchange-defective mutants [14]. Balancer heterozygotes also do not cause a delayed response to DSBs or increase in the number of PCH2 foci as observed in DSB repair and exchange-defective mutants. Therefore, two pathways probably lead into a *pch2*-dependent checkpoint: a *mei-218*-dependent pathway involving the early function of DSB repair proteins and a *mei-218*-independent pathway involving the structure of chromosome axes.

Of the two pathways in Drosophila, the pachytene checkpoint in other organisms has similarities to the *mei-218*-independent pathway involving chromosome structure. Heterozygous inversions and translocations induce a pachytene delay, suggesting a model in which the pachytene checkpoint can respond to breaks in axis continuity between paired homologs. The *C. elegans* pachytene checkpoint also monitors meiotic chromosome structure since a defect in a SC-nucleating “pairing center” triggers a Pch2-dependent response [16]. Similarly, the budding yeast pachytene checkpoint has been proposed to monitor SC-dependent events that may involve the relationship between recombination complexes and chromosome axes [41]–[43]. Therefore, a common feature of the pachytene checkpoints in these organisms is their sensitivity to the axis continuity between paired homologs with the main difference being SC-dependent defects (yeast and nematodes) *versus* SC-independent axis defects (Drosophila). Interestingly, both yeast Pch2 and mouse Trip13/Pch2 have been proposed to have a checkpoint-independent role in the organization of chromosome axis proteins [43], [44]. We do not know as of yet, however, if this is related to the sensitivity of paired axes at the Drosophila pachytene checkpoint, although it is tempting to suggest such a model.

**Figure 7:**
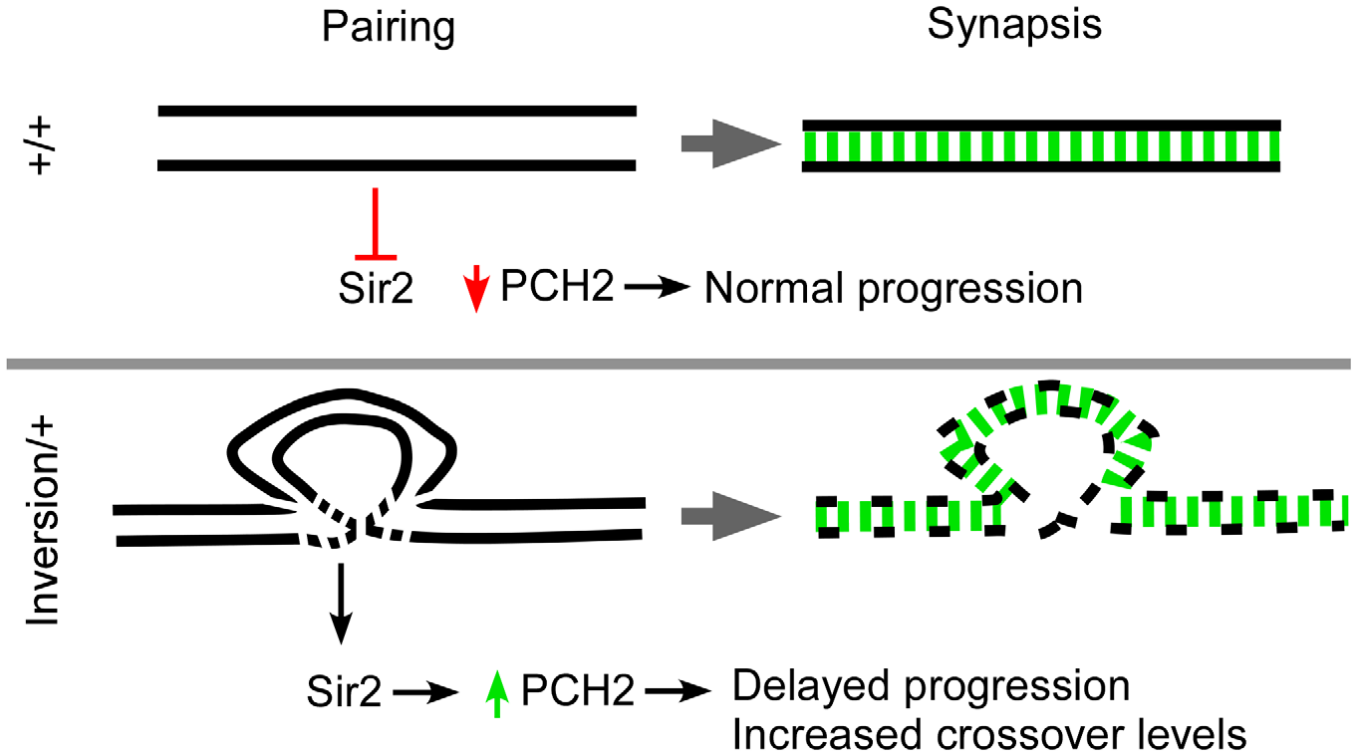
Model for pachytene checkpoint activity. Homologous axes are denoted as black lines and synapsis is in green. In wild-type cells (*+/+*), successful pairing between homologous axes causes PCH2 to degrade or inhibits its production prior to mid-pachytene. Rearrangements cause breaks in homology which can result in localized defects in the axis structure that destabilize over time. The axis defects allow Sir2 to modulate PCH2 levels by inhibiting its degradation or directly promoting its stability. The persistence of PCH2 activity in pachytene is sufficient to induce a delay in oocyte selection and increase the chance of DSBs becoming crossovers. Not shown is a second *mei-218*-dependent pathway involving early functions of DSB repair proteins that can also enhance pachytene checkpoint activity and mediate prolonged PCH2 expression [14].

Pachytene checkpoint activity in budding yeast is associated with prolonged Pch2 expression that requires Sir2 [15]. As in budding yeast, Drosophila *sir2* mutants are defective in the pachytene checkpoint and our overexpression studies suggest Sir2 is also required for the prolonged expression of PCH2. These results provide evidence for an evolutionarily conserved role of Pch2 and Sir2 in monitoring changes in chromosome structure during meiotic prophase from yeast to a metazoan (Figure 7). Drosophila may have evolved an additional *mei-218*-dependent pachytene checkpoint, not shared with yeast and nematodes, which is sensitive to DSB repair complexes.

### Chromosomal rearrangements induce *pch2*-dependent interchromosomal effects on crossing over

The effect of inversion heterozygosity on the frequency of crossing over has been known since the work of Sturtevant in 1919. Most often these intrachromosomal rearrangements cause an interchromosomal increase in recombination levels. Exhaustive work has been carried out investigating the interchromosomal effect and several models have been proposed in order to account for the increase in crossing over [11]. The most recent and generally accepted model was last described by Lucchesi and Suzuki in 1968 who proposed a timing model where pairing and crossover formation are coupled during the pachytene stage of prophase [11]. They suggested that when the pairing process between one set of homologs is perturbed or delayed by chromosome rearrangements, pachytene was lengthened and the opportunity to make crossovers was prolonged. We propose a modified version of the timing model where breaks in homology cause disruptions in the axis structure, resulting in a checkpoint-mediated delay (Figure 7).

The timing model proposed by Lucchesi and Suzuki predicts that a factor exists which controls the timing of meiotic prophase and can affect the level of exchange [11]. The pachytene checkpoint may regulate this timing mechanism. Although *pch2* is dispensable for crossing over in a wild-type background, it is required for most of the residual crossovers that occur in recombination-defective mutants. *pch2* is also required for most of the interchromosomal effect and pachytene delays observed in inversion heterozygotes. To our knowledge, *pch2* is the first example of a gene in Drosophila required for the interchromosomal effect that is not required for crossing over in general. Prolonged PCH2 expression may facilitate the formation of more crossovers by simply delaying a pachytene transition and extending the crossover determination phase, thereby allowing more crossover sites to be selected. An alternative explanation is that *pch2*, while not required for crossover formation in wild-type, is required for a crossover mechanism active only in axis-defective situations. Since the interchromosomal effect is not mediated by an increase in DSBs, PCH2 most likely increases the chance of DSBs becoming crossovers at the expense of noncrossovers.

### PCH2 function, localization, and mechanism of checkpoint activity

Drosophila PCH2 localizes to peri-nuclear foci in zygotene and early pachytene cells and is rapidly degraded or no longer made at mid-pachytene. In mutants in which pachytene delays are observed, PCH2 expression persists longer than in wild-type. The observation that overexpressing PCH2 induces effects on both timing and crossover levels indicates prolonged PCH2 expression is necessary and sufficient for the pachytene checkpoint's downstream effects. Since the duration of early pachytene correlates with the domain of PCH2 expression, we suggest that degradation of PCH2 turns off checkpoint activity and allows progression through pachytene, which ends the crossover determination phase (Figure 7).

We observed PCH2 localization to the outside of the nuclear envelope. These results were surprising considering the effect a *pch2* mutation has on nuclear events like crossing over. While we cannot rule out the possibility that a small undetectable fraction of PCH2 protein enters the nucleus and interacts with the chromosomes, PCH2 may indirectly affect nuclear events by facilitating interactions between the chromosomes and the nuclear envelope. In budding yeast, the pachytene checkpoint requires the localization of Pch2 to the nucleolus [15]. Therefore, like budding yeast, PCH2 in Drosophila may mediate the pachytene checkpoint at a distance from the recombination sites. Intriguingly, the nuclear envelope has been linked to several cellular processes relevant to meiotic recombination, including homolog pairing and DSB repair [45]–[48]. In *C. elegans,* the pairing of homologous chromosomes first requires the relocation of chromosomal regions known as pairing centers to the nuclear envelope [45]. Chromosome deficiencies that remove the pairing center impair relocation, homolog pairing and synapsis as well as trigger a *pch2*-dependent response [16]. Therefore, it is possible that in other organisms, the nuclear envelope has a conserved role in transducing pachytene checkpoint effects.

## Materials and Methods

### Drosophila strains

Drosophila stocks and crosses were maintained on a standard medium at 25°C. The following mutant alleles were used unless otherwise noted- *ord^10^* [20], *c(2)M^EP^*, *pch2^EY01788a^ (pch2^EY^)*, *c(3)G^68^* [18], *hdm^g7^*, *mei-218^1^*, *rec^1^and rec^2^* [49], *Ercc1^X^* [30], *mei-9^a^*, *spn-A^1^*, *spn-B^Bu^*, *sir2^17^* [38], and *mei-W68^4572^*. The deficiency *Df(2L)BSC245* deletes cytological bands 33F3-34A9, which includes the *sir2* locus. *T(2;3)DP^77^* and *T(2;3)dp^D^* translocations were obtained from the Bloomington Stock Center. *T(2;3)DP^77^* breakpoints are at 26E-27A on the 2^nd^ and 85C on the 3^rd^. *T(2;3)dp^D^* breakpoints are at 25A on the 2^nd^ and 95B–D on the 3^rd^. The *T(2;3)lt^X16^* translocation has breakpoints at 40 (heterochromatin) on the 2^nd^ and 95A3 on the 3rd and was obtained from B. Wakimoto [50].

### Genetic techniques

X-chromosome nondisjunction was assayed by crossing females to *y w/Y^BS^* males. The frequency of X-chromosome nondisjunction is calculated as 2(Bar females + Bar^+^ males)/[2(Bar females + Bar^+^ males) + Bar^+^ females + Bar males]. To estimate wild-type X chromosome crossing over frequency, *y/y pn cv m f • y^+^* female flies were crossed to *C(1:Y)1, v f B: [+]; C(4)RM, ci ey* males, and X chromosome recombinants were scored in males. Second chromosome crossing over was assayed by crossing *al dp b pr cn/+* females to *al dp b pr cn/CyO* males and scoring for recombinants among the *Cy^+^* progeny. P-values were calculated using the Fisher's exact test.

### Cytology and immunofluorescence

For immunolocalization experiments, females were aged at room temperature for about 16 hours and ovaries were dissected and fixed using the “Buffer A” protocol [51]. The antibody to γ-H2AV was described by Mehrotra et al. [28] and used at a 1∶500 dilution. Additional primary antibodies included mouse anti-C(3)G antibody used at 1∶500 [18], rabbit anti-C(2)M antibody used at 1∶400 [52], a combination of two mouse anti-Orb antibodies (4H8 and 6H4) used at 1∶100 [53], mouse anti-Lamin antibody developed by Fisher, P.A. used at 1∶800, and a rat anti-HA antibody (Roche) used at 1∶15.

The secondary antibodies were Cy3 labeled goat anti-rabbit (Jackson Labs) used at 1∶250, Cy3 labeled goat anti-rat (Jackson Labs) used at 1∶100 and Alexa fluor 488 goat anti-mouse (Invitrogen) used at 1∶100. Chromosomes were stained with Hoechst 33342 at 1∶50,000 (10 mg/ml solution) for seven minutes at room temperature. Images were collected using a Leica TCS SP2 confocal microscope with a 63X, N.A. 1.3 lens. In most cases, whole germaria were imaged by collecting optical sections through the entire tissue. These data sets are shown as maximum projections. The analysis of the images, however, was performed by examining one section at a time.

### Counting the frequency of the two-oocyte phenotype and calculating P-values

The oocytes were observed using an anti-C(3)G antibody. In some cases, such as when C(3)G staining was not visible, anti-ORB staining was used to identify the oocyte(s). A cell was scored as an oocyte if complete SC filaments were clear and distinct or by a concentration of ORB staining in the cytoplasm of a cell [54]. P-values were calculated using the Fisher's exact test. The P-value from the test compares the ratio of one-oocyte to two-oocyte cysts that were observed in two genotypes.

### Counting γ-H2AV foci in repair-proficient and repair-defective backgrounds

The γ-H2AV foci were counted from germaria where the foci were clear and distinct. Foci numbers in wild-type were at a maximum in region 2a (early pachytene) and few foci were visible by region 2b (mid pachytene). Therefore, to compare foci numbers in different genotypes, we used a method that calculates all cysts with γ-H2AV foci, averaging the amount in each pair of pro-oocytes. We compared the average foci in all the pro-oocytes or oocytes of each germarium, starting with the youngest cysts at the anterior end, by examining a full series of optical sections.

For counting γ-H2AV foci in repair-defective backgrounds, ORB staining was used to identify oocytes in region 3. The foci were counted from germaria where the foci were clear and distinct. The foci were counted manually by examining each section in a full series of optical sections containing complete pro-oocyte nucleus.

### Plotting γ-H2AV foci as a function of relative cyst age

Since the position of a cyst in the germarium is only a rough estimate of its meiotic stage, the foci were first counted in all the pro-oocytes/oocytes (identified by C(3)G staining) in the germarium. The meiotic stage of each pro-oocyte was then normalized according to the relative position of the entire cyst within the germarium since the relative position is more important than absolute position. The pro-oocytes from 13 wild-type germaria, 4 *FM7/+*, 4 *CyO/+*, 5 *FM7/+; CyO/+*, 5 *spn-B^Bu^*, and 4 *sir2^17^/Df; spn-B^Bu^* were arranged according to their relative age. The average number of γ-H2AV foci per pro-oocyte at each stage was then calculated and plotted as a function of relative cyst age.

### Construction of PCH2 transgenes

The annotated coding region of *pch2* was obtained from Flybase and amplified off the *pch2* cDNA clone LD24646 [55] by PCR. The coding region of *pch2* was then cloned into the Gateway® pENTR^TM^4 vector (Invitrogen). An LR ‘clonase’ reaction was then performed to recombine *pch2* into the ppHW destination vector (Invitrogen) that contains 3 copies of an N-terminus HA-tag under the control of an inducible UASP promoter. The construct was injected into fly embryos by Model System Genomics at Duke University. To express the transgenic lines, they were crossed to flies expressing Gal4 using either the NGT (*P[Gal4-nos.NGT]40*) [35] or MVD1 (*P[Gal4::VP16-nos.UTR]MVD1*) drivers [37].

### Counting PCH2 foci

The HA-PCH2 foci were counted from germaria where the foci were clear and distinct. We counted the average foci surrounding nuclei in all the cysts at region 2 and region 3 of each germarium by examining a full series of optical sections.

## Supporting Information

Figure S1 Transgenic rescue of *pch2*-dependent delay. Transgenic *pch2* expressed by the *NGT* driver restored the high frequency of the two-oocyte phenotype found in *FM7/+* heterozygotes. The two oocyte phenotype was assayed by C(3)G staining and an asterisk is located above each bar when P-value was <0.05 compared to wild-type. The number of cysts counted is at the bottom of each bar.
(0.18 MB TIF)

Figure S2 PCH2 localization in *mei-218* mutants. MVD1-driven PCH2 expression persists into region 2b and region 3 in a mei-218 mutant. To the right is shown a single section of an early pachytene oocyte with PCH2 foci adjacent to the DNA stain, indicating that mei-218 has no effect on the localization pattern of PCH2 within a cell.
(1.76 MB TIF)

Figure S3 C(2)M Expression by the *P(UAS:c(2)M^3XHA^)* transgene in wild-type and *sir2* mutants. Germaria are stained with anti-HA to detect transgenic *MVD1*-driven *UASP:c(2)M*. In wild-type (*MVD1 UASP:c(2)M/+*), transgenic C(2)M staining is present in the pro-oocytes throughout the germarium. In *sir2* mutants, transgenic C(2)M staining is as robust as in wild-type, indicating the transcription of *UASP*-driven genes is not affected in this background. The images are a maximum projection of all sections through the germaria. Scale bar is 10 µM.
(2.64 MB TIF)

Table S1 X-Chromosome nondisjunction in *Ercc1, pch2* and *sir2* mutants.
(0.03 MB DOC)
