# Supplementary figures and images for: Chromosome Axis Defects Induce a Checkpoint-Mediated Delay and Interchromosomal Effect on Crossing Over during Drosophila Meiosis
Source: PLoS Genet. 2010 Aug 12;6(8):e1001059. doi: 10.1371/journal.pgen.1001059 (PMC2920846; doi:10.1371/journal.pgen.1001059)

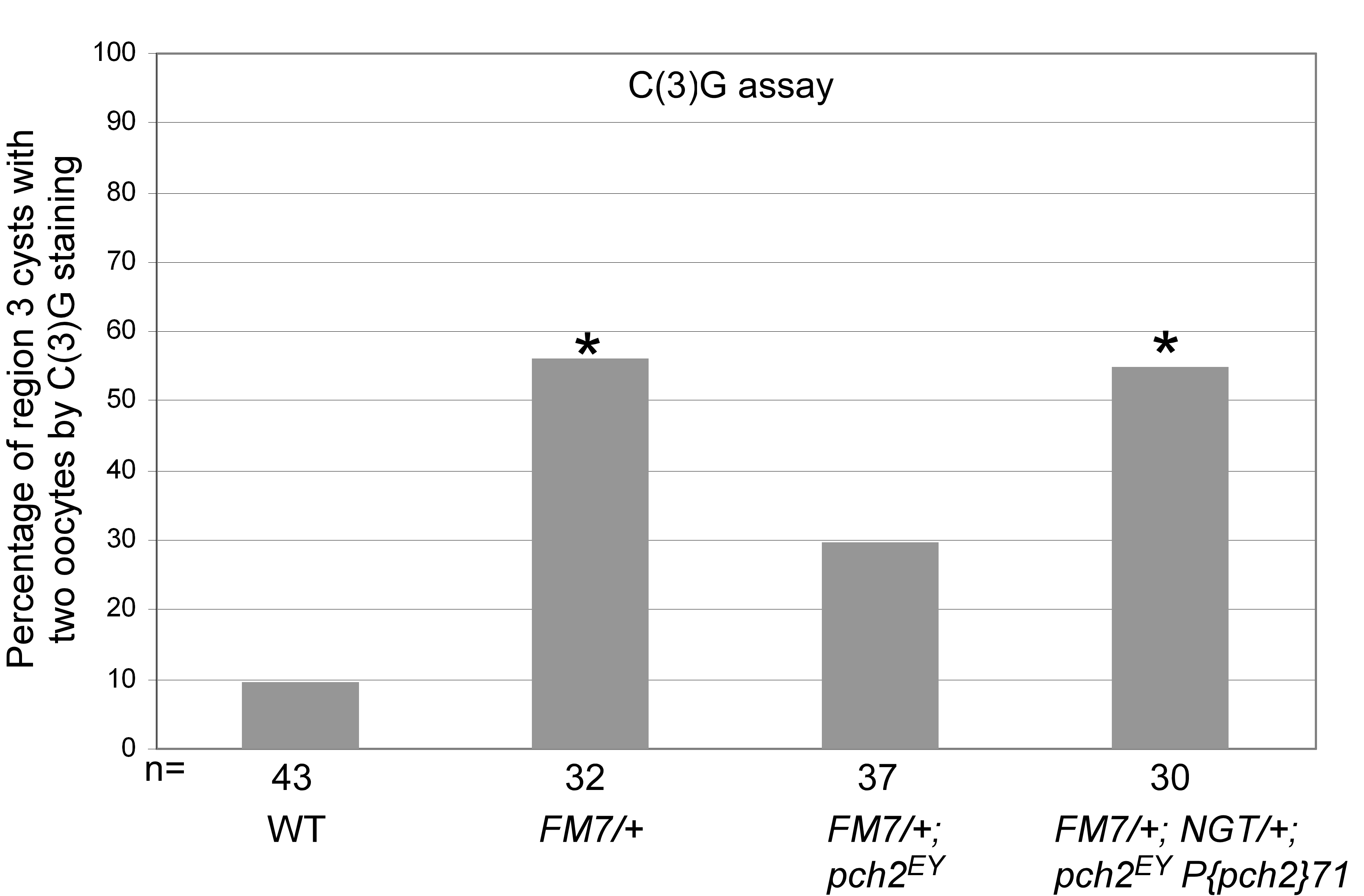

Supplement: Figure S1 — Transgenic rescue of pch2-dependent delay. Transgenic pch2 expressed by the NGT driver restored the high frequency of the two-oocyte phenotype found in FM7/+ heterozygotes. The two oocyte phenotype was assayed by C(3)G staining and an asterisk is located above each bar when P-value was <0.05 compared to wild-type. The number of cysts counted is at the bottom of each bar. (0.18 MB TIF) [file pgen.1001059.s001.tif]

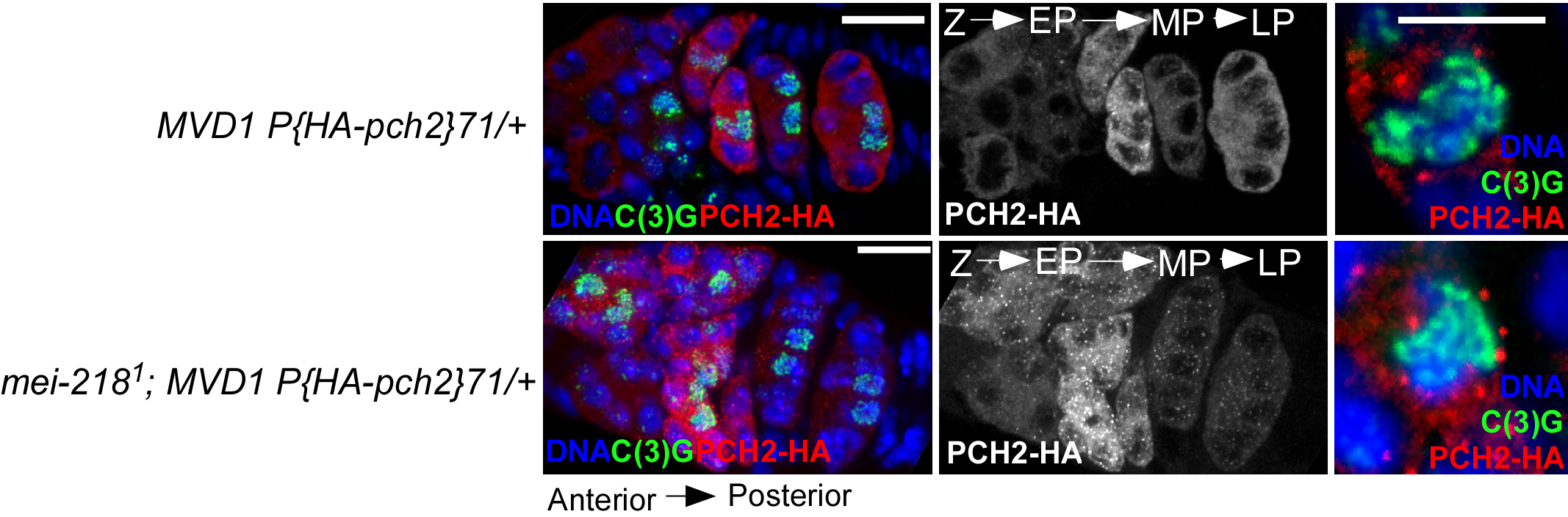

Supplement: Figure S2 — PCH2 localization in mei-218 mutants. MVD1-driven PCH2 expression persists into region 2b and region 3 in a mei-218 mutant. To the right is shown a single section of an early pachytene oocyte with PCH2 foci adjacent to the DNA stain, indicating that mei-218 has no effect on the localization pattern of PCH2 within a cell. (1.76 MB TIF) [file pgen.1001059.s002.tif]

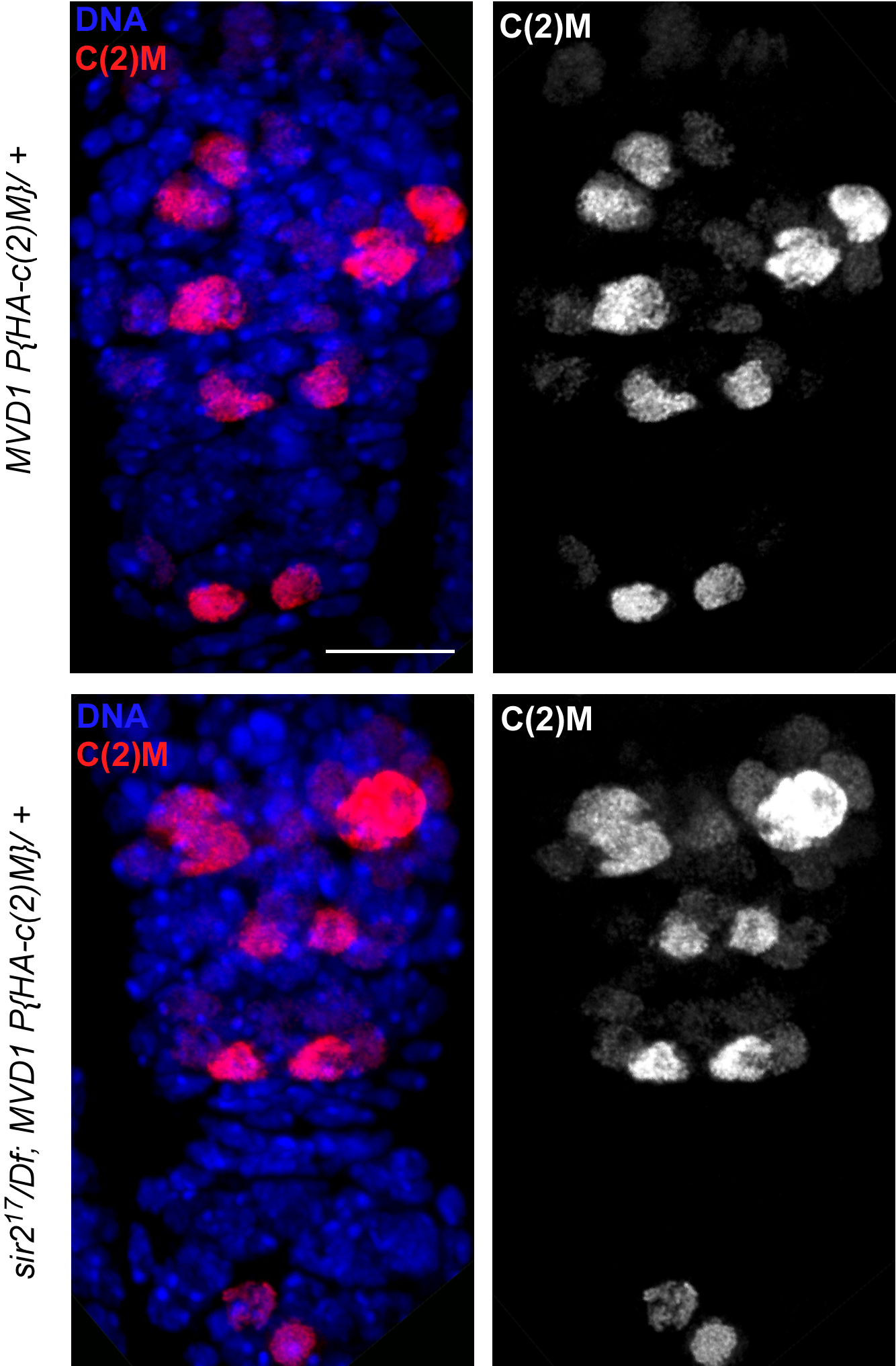

Supplement: Figure S3 — C(2)M Expression by the P(UAS:c(2)M3XHA) transgene in wild-type and sir2 mutants. Germaria are stained with anti-HA to detect transgenic MVD1-driven UASP:c(2)M. In wild-type (MVD1 UASP:c(2)M/+), transgenic C(2)M staining is present in the pro-oocytes throughout the germarium. In sir2 mutants, transgenic C(2)M staining is as robust as in wild-type, indicating the transcription of UASP-driven genes is not affected in this background. The images are a maximum projection of all sections through the germaria. Scale bar is 10 µM. (2.64 MB TIF) [file pgen.1001059.s003.tif]
