# Supplementary material for: Chromosome Axis Defects Induce a Checkpoint-Mediated Delay and Interchromosomal Effect on Crossing Over during Drosophila Meiosis
Source: PLoS Genet. 2010 Aug 12;6(8):e1001059. doi: 10.1371/journal.pgen.1001059 (PMC2920846; doi:10.1371/journal.pgen.1001059)
Supplement: Table S1 — X-Chromosome nondisjunction in Ercc1, pch2 and sir2 mutants. (0.03 MB DOC) [file pgen.1001059.s004.doc]

Table S 1. X-Chromosome nondisjunction in *Ercc1,* *pch2* and *sir2* mutants

|  | X-chromosome nondisjunction | |
| --- | --- | --- |
| **Genotype** | **% X-ND** | **N**^a^ |
| Wild-type | 0.1 | 2440 |
| *pch2^EY^* | 0.3 | 2098 |
| *Ercc1^X^* | 13.6 | 353 |
| *Ercc1^X^; pch2^EY^* | 30.0 | 300 |
| *sir2^17^/Df* | 0.2 | 632 |

^a^ N= total flies counted
